# Neurodegeneration and microtubule dynamics: death by a thousand cuts

**DOI:** 10.3389/fncel.2015.00343

**Published:** 2015-09-09

**Authors:** Jyoti Dubey, Neena Ratnakaran, Sandhya P. Koushika

**Affiliations:** ^1^Department of Biological Sciences, Tata Institute of Fundamental ResearchMumbai, India; ^2^InStemBangalore, India

**Keywords:** microtuble stability, Alzheimer's disease, Parkinson disease, dying back, hyperstable microtubules, microtubule signaling hubs

## Abstract

Microtubules form important cytoskeletal structures that play a role in establishing and maintaining neuronal polarity, regulating neuronal morphology, transporting cargo, and scaffolding signaling molecules to form signaling hubs. Within a neuronal cell, microtubules are found to have variable lengths and can be both stable and dynamic. Microtubule associated proteins, post-translational modifications of tubulin subunits, microtubule severing enzymes, and signaling molecules are all known to influence both stable and dynamic pools of microtubules. Microtubule dynamics, the process of interconversion between stable and dynamic pools, and the proportions of these two pools have the potential to influence a wide variety of cellular processes. Reduced microtubule stability has been observed in several neurodegenerative diseases such as Alzheimer's disease (AD), Parkinson's disease (PD), Amyotrophic Lateral Sclerosis (ALS), and tauopathies like Progressive Supranuclear Palsy. Hyperstable microtubules, as seen in Hereditary Spastic Paraplegia (HSP), also lead to neurodegeneration. Therefore, the ratio of stable and dynamic microtubules is likely to be important for neuronal function and perturbation in microtubule dynamics might contribute to disease progression.

## Introduction

Neurons receive information and relay it along axons, to other neurons or muscles, through structures known as synapses. Neurodegeneration refers to the progressive loss of structure and/or function of neurons, often beginning at the synaptic distal ends of axons, a phenomenon termed as “dying-back neuropathy” (Goto et al., 2009; Dadon-Nachum et al., 2010). Neurodegeneration in humans can cause a variety of symptoms depending on the class of neurons affected and can lead to fatal outcomes. Some widely studied neurodegenerative diseases include Alzheimer's disease (AD), Parkinson's disease (PD), Huntington's disease (HD), and Amyotrophic Lateral Sclerosis (ALS). These, and other less well studied neurodegenerative diseases, exhibit a broad range of clinical symptoms, which nonetheless share several common pathological features (Skovronsky et al., 2006; Glass et al., 2010; Arnold et al., 2013; Baird and Bennett, 2013). One prominent cellular feature is the toxic aggregation of proteins that inhibit the protein quality control and the ubiquitin-proteasome machinery of the neuron (Skovronsky et al., 2006; Arnold et al., 2013; Takalo et al., 2013). Other common characteristics include inflammatory responses (Glass et al., 2010), impaired ER calcium homeostasis (Paschen and Mengesdorf, 2005), increased oxidative stress (Ischiropoulos and Beckman, 2003), and microtubule defects (Baird and Bennett, 2013). It is essential to recognize common underlying features that permit degeneration of neurons to understand the most frequent mechanisms contributing to disease progression. Such an understanding can potentially lead to better therapeutic agents that can retard progression of the most debilitating symptoms associated with neurodegeneration.

Microtubules have often been thought to participate in neurodegenerative diseases through their well-established role in long-distance cargo transport. Examples include neurodegenerative diseases such as AD, HD, and several tauopathies that show compromised microtubule-dependent axonal transport in disease models (extensively reviewed in Garcia and Cleveland, 2001; Goedert and Jakes, 2005; De Vos et al., 2008; Baird and Bennett, 2013; Franker and Hoogenraad, 2013; Hinckelmann et al., 2013; Millecamps and Julien, 2013; Beharry et al., 2014; Encalada and Goldstein, 2014). However, these polymers play important roles in many aspects of neuronal cell biology that include establishing and conserving neuronal polarity (Baas et al., 1988, 1989; Craig and Banker, 1994; Witte et al., 2008), maintaining neuronal morphology (Jacobs and Stevens, 1986; Jaworski et al., 2009; Liu and Dwyer, 2014) and modulating signaling events (Janmey, 1998; Wittmann and Waterman-Storer, 2001; Bounoutas et al., 2011; Dent and Baas, 2014). In several neurodegenerative diseases, that include sporadic rather than familial cases, we know little about the causes of disease onset. We propose that disruption of microtubule dynamics may be a key mechanism contributing to neurodegeneration, since an alteration in dynamics can affect a number of the roles mentioned above. Therefore, modulating microtubule dynamics might help in retarding the progress of neurodegenerative diseases.

## Microtubules contribute to neuronal polarity

**Figure 1:**
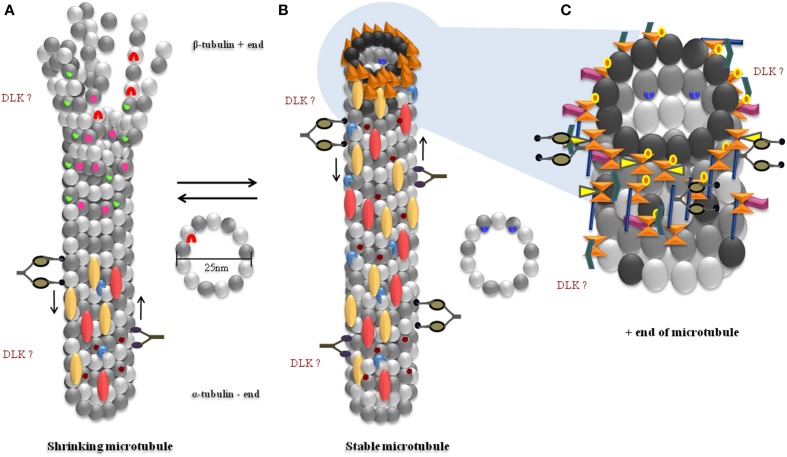
**Proteins and modifications associated with unstable and stable microtubules. (A)** Shrinking microtubules disassemble from their plus ends, lose their MAPs, are not acetylated but are tyrosinated. LRRK2 binds to the luminal side of β-Tubulin and prevents acetylation of microtubules. **(B)** Stable microtubules have a large complement of proteins associated with them, are not tyrosinated, are acetylated and have GTP-capped ends with multiple proteins. **(C)** Plus-ends of microtubules have several +TIP proteins. Several bind to the plus end binding EB proteins and GTP bound β-Tubulin. The precise location of DLK binding on microtubules is unknown. α-Tubulin (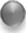), β-Tubulin (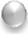), GTP bound β-Tubulin (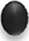), α/β heterodimer (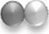), Kinesin motor (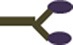), Dynein motor (
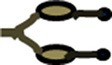
). Present on less stable microtubules: Tyrosination (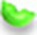), Gαs (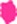), LRRK2 (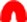). Present on stable microtubules: MAP1 (axon and dendrites) (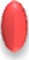), TAU (axon) (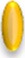), MAP2 (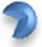), Acetylation (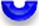), Gβγ (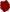), +end binding proteins (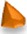). Proteins present on the +end or fast growing end of microtubules (+TIPs): EBP1/2/3 (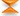), CLIP170 (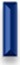), CLASPS (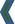), APC (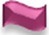), RHO GEF2 (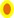), MACF (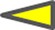).

A critical aspect of neuronal function depends on establishing the polarity between the axonal and somatodendritic domains of the neuron (Craig and Banker, 1994). Microtubules are inherently polarized structures, with the α-tubulin subunits present at the slow growing “minus end” and the β-tubulin subunits at the fast growing “plus end” of the tube (Allen and Borisy, 1974) (Figure 1). Microtubules in the axon of a neuron display uniform polarity, with their plus ends oriented toward synapses, whereas those in the dendrites display mixed polarity (Burton and Paige, 1981; Baas et al., 1988, 1989; Dombeck et al., 2003; Stepanova et al., 2003, 2010). Microtubules contribute to the overall polarity by being involved in axon specification, an important early event in establishing neuronal polarity (Burton and Paige, 1981; Heidemann et al., 1981; Witte et al., 2008; Hoogenraad and Bradke, 2009). During this process, microtubules in a single neurite reorganize their mixed polarity orientation to exclusively plus-end distal microtubules, thus breaking cellular symmetry and marking distinct axonal and dendritic domains (Baas et al., 1989; Horton and Ehlers, 2003). This intrinsic polarity of microtubules plays a critical role in the precise trafficking of a variety of cargo within a neuron (Zheng et al., 2008; Maday et al., 2014). Disorganization of microtubule polarity can result in incorrect localization of cargo. For example, in *Drosophila*, loss of the Dynein motor or microtubule associated LIS1 (both implicated in lissencephaly), results in axons containing both plus- and minus end-distal microtubules, leading to mis-trafficking of dendritic proteins into axons (Liu et al., 2000; Vallee and Tsai, 2006; Zheng et al., 2008; Reiner, 2013). Similarly, in *C. elegans*, disruption of microtubule polarity causes mislocalization of an axonal Kinesin-3 motor, synaptic vesicles (Maniar et al., 2012), and dense core vesicles (Goodwin et al., 2012) to dendrites, leading to non-specific targeting of axonal proteins. Recovery from complete axon loss after axotomy, in cultured neurons, requires microtubule reorganization and polarity reversal in one of the remaining neurites to re-specify the axon (Gomis-Rüth et al., 2008). An *in vivo* study using *Drosophila* shows that this change in microtubule polarity, and consequent re-specification of the axon, is preceded by an increase in the polymerization of microtubules (Stone et al., 2010). In contrast, axon specification is preceded by an increase in stability of microtubules in a single neurite (Witte et al., 2008). Additionally, increasing microtubule stability by Taxol treatment can result in the formation of multiple neuronal processes that exhibit characteristics of a typical axon, viz. localization of axonal markers Tau and synapsin-1 (Witte et al., 2008). These studies show that microtubule dynamics contribute to changes in microtubule orientation and consequently some aspects of neuronal polarity. Altered protein distribution arising from the changes in polarity can be detrimental for the neuron, thereby contributing to disease symptoms.

## Microtubule dynamics maintain neuronal morphology

Neuronal morphology and formation of specific connections to both its pre-and post-synaptic partner cells are critical for neuron function (Morales et al., 2002; Chen et al., 2006; Lewcock et al., 2007). Several studies also show that the morphology of the neuron can be influenced by microtubules. For instance, specific mutations in the *C*. *elegans mec-7* β-tubulin lead to ectopic neurite outgrowth that is suppressed upon treatment with the microtubule-destabilizing drug colchicine (Kirszenblat et al., 2013). Hyperactivation of the Notch signaling pathway is also able to regulate axonal morphology, resulting in thicker neurites, fewer branches, and loss of synaptic varicosity, thought to arise from the observed hyperstabilization of microtubules (Ferrari-Toninelli et al., 2008; Bonini et al., 2013). Such hyperstabilization likely arises from the Notch-induced increase in acetylation and polyglutamylation of α-tubulins, both of which are markers of stable microtubules (Ferrari-Toninelli et al., 2008). Notch is also thought to increase microtubule stability by reducing the expression of the microtubule severing enzyme Spastin (Ferrari-Toninelli et al., 2008). The downstream effectors that mediate such effects of Notch signaling have not been identified. However, a downstream kinase Abl known to interact with microtubules (Miller et al., 2004) and required for axon growth (Giniger, 1998), is a potential candidate that could mediate Notch dependent microtubule effects. These studies suggest that hyper-stable microtubules might be detrimental to neuronal morphology.

Likewise, unstable microtubules also influence neuronal morphology. Unstable microtubules and microtubule-actin interactions are essential for formation of neuronal branches (Dent and Kalil, 2001). Plus-end-binding proteins (+TIPs), such as CLASPs, APC, and MACF that bind to polymerizing microtubules and stabilize it, are required for the microtubule-actin interactions that promote axon elongation or branching (Leung et al., 1999; Zhou et al., 2004; Kornack and Giger, 2005; Watanabe et al., 2009). Therefore, circumstances that elevate dynamic microtubule pools promote axon branching. For example, inhibition of microtubule stabilizing proteins such as Tau result in increased axonal branching (Yu et al., 2008). In addition, the microtubule-associated ubiquitin ligase Phr1 mutants, in mouse, and zebrafish, have axons with sharp kinks, abnormally bent growth cones and mistargeting of motor neurons to inappropriate tissues (Lewcock et al., 2007; Hendricks and Jesuthasan, 2009). One of these studies shows that these axonal abnormalities were greatly reduced by taxol-induced stabilization of microtubules, suggesting that axonal morphology itself might depend on microtubule stability (Lewcock et al., 2007). This hypothesis is also supported by the observed change in dendrite morphology from spine-like to more filopodia-like structures upon destabilization of microtubules in hippocampal CA1 neurons using nocadazole (Jaworski et al., 2009).

All of the above studies also describe cellular processes that are disrupted when microtubule stability is altered; such as axon path-finding and innervation (Lewcock et al., 2007), synaptic plasticity (Jaworski et al., 2009) and regenerative capacity after injury (Kirszenblat et al., 2013). These studies suggest that disproportionate levels of hyper-stable or very unstable microtubules are both detrimental to neuronal morphology.

## Microtubules are highways for cargo transport

Microtubules serve as the sub-cellular roads for long-distance transport where they act as “tracks” on which motor proteins such as kinesin and dynein transport multiple organelles and macromolecules (Gunawardena and Goldstein, 2004; Hirokawa and Noda, 2008). The Kinesin family of motors move toward the plus ends of microtubules while Dynein motors walk toward the minus end (Vale et al., 1985a,b; Paschal and Vallee, 1987; Wade, 2009) (Figure 1). Microtubule-mediated fast axonal transport occurs at a speed of ~50–200 mm/day and delivers axonal cargo such as Amyloid Precursor Protein (APP), synaptic proteins, synaptic vesicles, and dense core vesicles (Maday et al., 2014). Microtubule-dependent slow axonal transport occurs at a speed of ~0.2–10 mm/day and carries cytoskeletal proteins such as Tubulin and cytosolic proteins such as Synapsin (Maday et al., 2014; Roy, 2014). Lack of fast axonal transport is known to cause developmental and functional defects in the motor neurons of *Drosophila* (Hurd and Saxton, 1996), in PLL axons of zebrafish (Lyons et al., 2008), in the spinal nerves and peripheral axons in mice (Warita et al., 1999), and in multiple neurons of *C*. *elegans* (Hall and Hedgecock, 1991).

**Figure 2:**
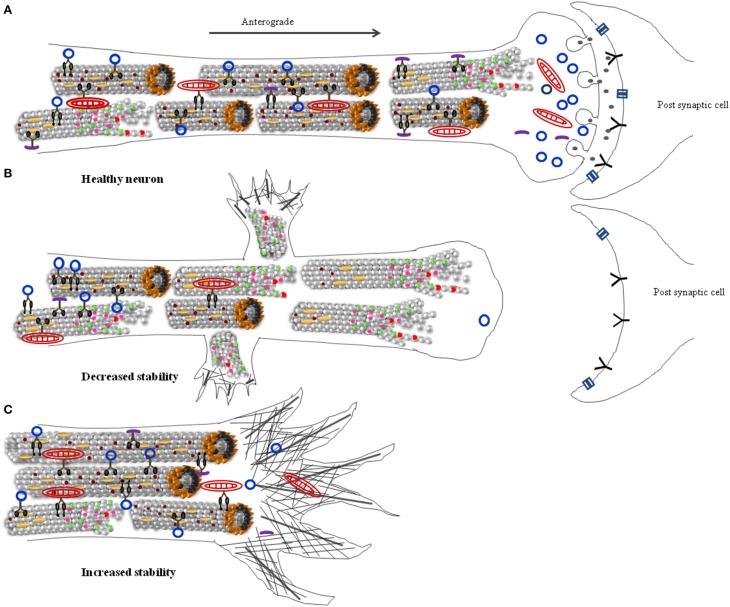
**Ratios of stable and dynamic microtubule alter neuronal structure and function. (A)** Healthy neurons have short and long, stable, and dynamic microtubules **(B)** Increased numbers of dynamic microtubules lead to increased neuronal branching, synapse retraction, and reduced axonal transport. This eventually can lead to dying-back neuropathy. **(C)** Hyperstable microtubules increase the diameter of the neuron, inhibit neurite outgrowth, and inhibit neuronal branching. Stable microtubule (
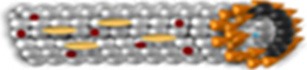
), depolymerzing microtubule (
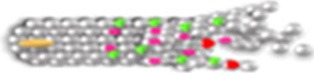
), microtubule associated proteins (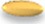), microtubule plus end binding proteins (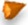), mitochondria (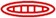), membranous cargo (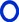), non-membranous cargo (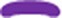), Kinesin motor (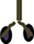), Dynein motor (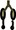), Actin filaments (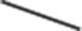), Actin bundles (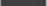), neurotransmitters (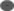), channels (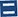), neurotransmitter receptors (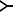).

Stable microtubules are an integral part of maintaining axonal transport. When cultured neurons are treated with Parkinsonism-inducing neurotoxin 1-methyl-4-phenylpiridinium (MPP+), there is reduction in microtubule dynamics, perhaps accounting, in part, for the observed mitochondrial accumulation along axons (Cartelli et al., 2010). Another study showed that hyper-dynamic microtubules occur very early, before observable impairment of axonal transport, in a mouse disease model of ALS (Fanara et al., 2007). Further, drug-induced stabilization of microtubules was able to restore normal axonal transport, suggesting that axonal transport defects in this model are a consequence of increased microtubule dynamics (Fanara et al., 2007) (Figures 2A,B). These examples suggest that altered microtubule dynamics are detrimental to axonal transport. This raises the intriguing possibility that several of the observed transport defects, in neurodegenerative diseases (De Vos et al., 2008; Millecamps and Julien, 2013), might arise at least in some instances from altered microtubule dynamics.

## Microtubules as regulators of signaling and gene expression

It has been suggested recently that neuronal microtubules, through their interactions with G-proteins (Wittmann and Waterman-Storer, 2001), plus-end-tracking proteins (+TIPs) (Akhmanova and Steinmetz, 2008) and minus-end-targeting proteins (-TIPs) (Akhmanova and Hoogenraad, 2015), might act as carriers of information (Dent and Baas, 2014) (Figure 1). For example, disruption of microtubules leads to activation of microtubule-bound RHGF-1, a PDZ Rho GEF, and causes retraction of a collateral neuronal branch in *C*. *elegans* (Chen et al., 2014). This RHGF-1 dependent remodeling of neuronal morphology was shown to depend on the MAPKKK, DLK-1 (Chen et al., 2014). This finding is consistent with localization of DLK at the tips of microtubules in cultured cortical neurons (Hirai et al., 2002). DLK activity is also necessary to initiate repair processes after axonal injury (Yan et al., 2009; Ghosh-Roy et al., 2010; Xiong et al., 2010; Chen and Chisholm, 2011), and it has been suggested that cytoskeletal damage alone is potentially sufficient to trigger DLK activity, perhaps following DLK's release from microtubules (Valakh et al., 2013, 2015). Depolymerization of microtubules by chemical treatment, as well as decrease in microtubule number through mutations in *mec-12* α-tubulin and *mec-7* β-tubulin, cause reduction in expression of proteins through the DLK/p38 MAPK pathway in the touch receptor neurons of *C. elegans* (Bounoutas et al., 2011). Thus, microtubule numbers and dynamics appear to regulate DLK-dependent signaling in neurons.

Other signaling molecules, such as the heterotrimeric Gsα, strongly interact with α-tubulin (Layden et al., 2008). Gsα activates tubulin GTPase, leading to destabilization of microtubules (Roychowdhury et al., 1999; Schappi et al., 2014). Likewise, binding of Leucine-rich repeat kinase 2 (LRRK2) also regulates stability of microtubules by modulating tubulin acetylation (Gandhi et al., 2008; Law et al., 2014). LRRK2 is known to participate in several signaling cascades that modulate neurite growth, vesicle trafficking, endocytosis, and autophagy, although it is unclear whether all these functions require the microtubule-bound form of LRRK2 (Berwick and Harvey, 2011). Thus, signaling and scaffolding molecules can control microtubule dynamics; and microtubules themselves could function as signaling hubs by sequestering kinases such as DLK and LRRK2.

## Microtubule assembly, stability, and dynamics

Despite the importance of microtubules, their assembly within neurons is poorly understood. Two models have been proposed for the origin of axonal microtubules: (i) microtubules are clipped off from the centrosome by the severing enzyme, Katanin, before entering the axon (Baas et al., 2005) and (ii) multiple nucleation centers are created along the neuronal process from the severing of existing axonal microtubules (Roll-Mecak and Vale, 2006). Tubulin oligomers transported along the axon via slow transport (Terada et al., 2000; Wang and Brown, 2002) are eventually likely to be incorporated into microtubules. Once assembled, microtubules are dynamic, with cyclic phases of both growth and shrinkage. This behavior of microtubules has been termed “dynamic instability” (Box 1). The plus ends of microtubules are much more dynamic than their minus ends (Walker et al., 1988).

Box 1 **Experimental methods to monitor dynamic microtubules**

A key insight into microtubule polymerization came in the 1980s when coexistence of growing as well as shrinking populations of microtubules were observed *in vitro* by combining biochemical methods, immunofluorescence, and electron microscopy. This process was termed “dynamic instability” and was further confirmed in cells using similar methods (Cassimeris et al., 1988; Mitchison and Kirschner, 1988; Okabe and Hirokawa, 1988; Sammak and Borisy, 1988).

After these early studies, imaging techniques carried out at the time scales of 2 s or faster have been critical in observing microtubule dynamics *in vivo* (Shelden and Wadsworth, 1993). A major advance in the field was the development of fluorescent speckle microscopy (FSM). Here low concentrations of fluorescently labeled tubulin subunits were incorporated into a microtubule (Waterman-Storer and Salmon, 1998). The speckles provided markers along microtubules whose positions did not change unless they grew or shrunk. This technique unambiguously permitted tracking assembly and disassembly of microtubules.

The identification of microtubule end binding EB proteins now conveniently allows investigators to track the growing ends of microtubules using EB-GFP fusions (Stepanova et al., 2003). However, this method only assesses growth of +ends and does not provide the detailed information obtained using FSM and hence gives an incomplete understanding of microtubule dynamics.

A recent advance combined an electrically tunable lens with fluorescent microscopy thus providing rapid focusing (Nakai et al., 2015) which allows temporal resolution within 10 ms and a spatial resolution of 40 nm making it an attractive tool to assess microtubule dynamics *in vivo*. Such newer technologies may allow us to effectively monitor microtubule dynamics in healthy and diseased neurons.

Neurons possess more stable microtubules compared to other cell types (Okabe and Hirokawa, 1988; Seitz-Tutter et al., 1988; Stepanova et al., 2003). These stable microtubules have half-lives of several hours and co-exist with dynamic microtubules with half-lives of several minutes (Li and Black, 1996; Conde and Cáceres, 2009). A cap of non-hydrolyzed GTP Tubulin subunits at the growing end of a microtubule (Figures 1B,C) is thought to protect against depolymerization and stabilize the plus ends of microtubules (Carlier and Pantaloni, 1981; Mitchison and Kirschner, 1988). Another group of proteins that interacts with the fast growing ends of microtubules are the “plus end-tracking proteins” or +TIPs which include the cytoplasmic linker proteins (CLIPs), the CLIP-associated proteins (CLASPs), Adenomatous polyposis coli protein (APC), the Microtubule-actin crosslinking factor (MACF) and the end-binding (EB) proteins (Akhmanova and Steinmetz, 2010) (Figure 1C). The EB proteins are now widely used to track growing ends of microtubules (Stepanova et al., 2003; Akhmanova and Steinmetz, 2010) (Box 1). Further, several +TIP proteins regulate different aspects of microtubule dynamics (Table 1), including promoting interaction of microtubule ends with actin and other cellular structures (Akhmanova and Steinmetz, 2010).

**Table 1:** **Function of microtubule (MT) associated plus-end tracking proteins (+TIPs)**.

**TIPs**	**Interacting molecule(s)**	**Function**	**Plus end association**	**References**
EB-1/2/3	MT, ER, other +TIPs	MT polymerization, MT stabilization	Plus end directed transport	Akhmanova and Hoogenraad, 2005; Honnappa et al., 2006
CLASPS	EB1, CLIP-170, MT, F-actin	MT stabilization, axon guidance	Recognize other +TIP proteins	Kodama et al., 2003; Lee et al., 2004; Lansbergen et al., 2006
CLIP-115/CLIP-170	MT, EB1 CLIP-170 interacts with vesicles, F-actin	MT stabilization	Recognize other +TIPs, co-assembly with Tubulin dimers	Diamantopoulos et al., 1999; Akhmanova and Hoogenraad, 2005
APC	EB1, F-actin	MT stabilization	Kinesin based transport	Groden et al., 1991; Shi et al., 2004; Mimori-Kiyosue et al., 2005; Chazaud and Rossant, 2006
MACF	F-actin, EB1	MT stabilization	Recognize other +TIPs, directly bind Tubulin	Sun et al., 2001
RHOGEF2/RHGF	F-actin, EB1	MT polymerization	EB1 dependent binding	Rogers et al., 2004
Dynein	MT	MT stabilization	Plus end directed transport, recognize other +TIPs	Valetti et al., 1999; Akhmanova and Hoogenraad, 2005
STIM	EB1, ER, MT	MT stabilization	Recognize other +TIPs, directly bind Tubulin	Grigoriev et al., 2008
MCAK	EB1 and MT	MT catastrophe, depolymerization	Recognize other +TIPs, directly bind Tubulin	Kline-Smith and Walczak, 2002
LIS1	Dynein, CLIP-170	MT stabilization	Recognizes dynein	Tanaka et al., 2004; Vallee and Tsai, 2006

Lengths of neuronal microtubules are also known to vary and are controlled by the severing enzymes Spastin and Katanin, that can cut microtubules throughout their length (McNally and Vale, 1993; Karabay et al., 2004; Yu et al., 2005, 2008). Katanin levels are higher throughout the axon during neuronal development but the enzyme is mostly restricted to the cell body once axons reach their targets (Karabay et al., 2004; Yu et al., 2005). Katanin is therefore likely to sever microtubules, generating nucleating centers or tubulin subunits required for the process of axon extension during neuronal development. On the other hand, Spastin is concentrated at sites where axons branch (Yu et al., 2008), consistent with the presence of shorter microtubules at such locations (Yu et al., 1994). Further, certain MAPs may regulate the severing properties of enzymes, for example microtubule-bound Tau renders the microtubules more resistant to severing by Katanin but not Spastin (Qiang et al., 2006; Yu et al., 2008). Together these processes lead to axonal microtubules of various lengths. In several organisms these lengths vary from one micrometer to over hundred micrometers long, averaging around 100 μm in vertebrate neurons (Bray and Bunge, 1981; Chalfie and Thomson, 1982; Letourneau, 1982; Yu et al., 2007). An increased proportion of unstable and short microtubules might lead to varied neuronal defects such as impaired axonal transport and increased branching (Figure 2B) (Dent et al., 2003; Yu et al., 2008).

## Role of tubulin subunits in microtubule dynamics

Mutations in α- and β-Tubulin subunits can themselves influence the stability and dynamics of microtubules. Both α and β-Tubulins have an N-terminal domain, an intermediate domain and a C-terminal domain. Residues of the N-terminal domain are important for protein folding and conformation and contain the guanine nucleotide binding region (Downing and Nogales, 1998; Löwe et al., 2001). The residues in the C-terminal domain bind both MAPs and motor proteins (Littauer et al., 1986; Löwe et al., 2001). In *C. elegans*, approximately fifty mutations have been mapped to the *mec-7* β-tubulin gene and several to the *mec-12* α-tubulin gene (Savage et al., 1989, 1994; Fukushige et al., 1999; Baran et al., 2010; Bounoutas et al., 2011; Kirszenblat et al., 2013; Hsu et al., 2014). A missense mutation in the intermediate domain of MEC-7 induces ectopic neurite outgrowth, causes mis-localization of synaptic vesicles and reduces regeneration after injury (Kirszenblat et al., 2013). A recent study in *C. elegans* has also shown that a missense mutation in the C-terminal of MEC-12 α-tubulin leads to unbundling of microtubules and an increased affinity to Dynein that likely leads to the observed cargo mis-trafficking defects (Hsu et al., 2014). In both the above studies, lesions in distinct tubulin domains share similar cellular phenotypes. Several mutations in tubulin genes of *C*. *elegans* and other model systems map to many different locations along the proteins and lead to a multiplicity of neuronal phenotypes (Table 2). As yet it is unclear whether mutations in specific tubulin domains are associated with a single or specific constellation of phenotypes. Nonetheless, since mutations in all three domains of the tubulins have been associated with neuronal defects (Table 2), each protein domains appears to be critical for microtubule function. The aspect of microtubule structure or function altered in these cases is not uniformly well documented.

**Table 2:** **Effect of Tubulin mutations on neuronal function**.

**Gene**	**Mutation**	**Mutation location**	**Model system**	**Phenotype**	**References**
TUBB3	R262H, R262C	Intermediate	Mouse	Defects in axon guidance	Tischfield et al., 2010
TUBB2B	E421K	C-terminal	Mouse	Axonal dysinnervation	Cederquist et al., 2012
TUBB2B	S172P	N-terminal	Rat	Impaired microtubule assembly, defective migration of cortical neurons	Jaglin et al., 2009
TUBB2B	F265L	Intermediate	Rat	Impaired microtubule assembly, defective migration of cortical neurons	Jaglin et al., 2009
MEC-7	M1I, P171L	N-terminal	*C. elegans*	Defective neuronal morphology and behavioral defects	Savage et al., 1994
MEC-7	F317I, V286D	Intermediate	*C. elegans*	Defective neuronal morphology and behavioral defects	Savage et al., 1994
MEC-7	P220S	Intermediate	*C. elegans*	Defective neuronal morphology	Kirszenblat et al., 2013
MEC-7	W101X	N-terminal	*C. elegans*	Reduced neuronal gene expression	Savage et al., 1994; Bounoutas et al., 2011
MEC-7	P243L	Intermediate	*C. elegans*	Reduced neuronal gene expression	Savage et al., 1994; Bounoutas et al., 2011
MEC-7	A393T	C-terminal	*C. elegans*	Reduced neuronal gene expression	Savage et al., 1994; Bounoutas et al., 2011
MEC-12	K40R	N-terminal	*C. elegans*	Reduced protofilament number and behavioral defects	Cueva et al., 2012

Specific mutations in a human β-tubulin, TUBB3, have been shown *in vitro* and in cultured cells to impair α/β heterodimer formation leading to lower stability of microtubules (Poirier et al., 2010). These and other β-tubulin mutations have been associated with malformations in cortical development (MCD) in human patients and cortical neuron migration and axonal guidance defects in mouse models (Poirier et al., 2007, 2010; Jaglin et al., 2009). Such studies show that mutations in α- and β-tubulin that alter microtubule dynamics can lead to neurodevelopmental defects. Mutations in tubulin genes have also been documented in patients with many types of neurodegenerative diseases, although their relationship to microtubule dynamics or stability is yet unclear (Table 3).

**Table 3:** **Tubulin mutations associated with neurodegenerative diseases**.

**Gene**	**Mutation**	**Mutation location**	**Disease References**	
TUBB2A	N247K	Intermediate	Cortical dysplasia	Cushion et al., 2014
TUBB3	T178M, E205K	N-terminal	Malformation of cortical development and neuronal migration defect	Poirier et al., 2010
TUBB3	A302V, M323V	Intermediate	Malformation of cortical development and neuronal migration defect	Poirier et al., 2010
TUBB4A	D249N	Intermediate	Leukodystrophy	Simons et al., 2013
TUBB4A	R2Q, T178R	N-terminal	Leukodystrophy	Miyatake et al., 2014
TUBB4A	R53Q	N-terminal	Hypomyelinating leukoencephalopathies	Miyatake et al., 2014
TUBA4A	W407X	C-terminal	ALS	Smith et al., 2014
TUBB4A	R2G, R53G	N-terminal	DYT4 dystonia dysphonia	Lohmann et al., 2013

## Post-translation modifications as markers of microtubule stability

The α and β-Tubulin subunits themselves are modified by tyrosination, acetylation, and polyamination (Hammond et al., 2008; Fukushima et al., 2009; Janke and Chloë Bulinski, 2011). Some of these post-translational modifications mark stable microtubules (Westermann and Weber, 2003; Peris et al., 2006, 2009; Ikegami and Setou, 2010). For instance, stable microtubules are usually detyrosinated and acetylated (Fukushima et al., 2009). Detyrosination reduces microtubule depolymerisation (Peris et al., 2009) and detyrosinated microtubules are enriched in axons suggesting that axons contain more stable microtubules compared to dendrites (Witte et al., 2008; Hammond et al., 2010). Studies performed in cultured rat sympathetic neurons show that tyrosinated microtubules are enriched in the most proximal and distal regions of growing axons (Baas and Black, 1990; Brown et al., 1992). Further, a single microtubule can contain both stable and labile domains differing in their tyrosinated and acetylated Tubulin content (Baas and Black, 1990; Brown et al., 1992, 1993). Recently, polyamination of α- and β-Tubulins by a transglutaminase was also shown to promote axonal microtubule stabilization (Song et al., 2013). Such studies suggest that Tubulin modifications can affect both local stability within a microtubule and stability of microtubules along the axon. These islands of modifications within a microtubule may act as local disassembly brakes when microtubules undergo depolymerization. Additionally, the stable and dynamic regions of a microtubule could act as binding hotspots for specific microtubule-associated proteins such as LRRK2 to less stable microtubules (Law et al., 2014) and the motor Kinesin-1 to stable microtubules (Reed et al., 2006).

## Effect of MAPs on microtubule dynamics

Much of the increased stability of microtubules in neurons is due to the presence of a large number of MAPs that shift the dynamics toward assembly and promote stability (Figure 1B). These include classical MAPs such as Tau (MAPT), MAP1a, MAP1b, and MAP2 as well as STOP (Stable Tubule Only Protein), Doublecortin, and the microtubule crosslinking proteins Plakins/Plectins (Chapin and Bulinski, 1992; Matus, 1994; Bosc et al., 1996; Horesh et al., 1999; Leung et al., 2002).

MAP2 and Tau are present exclusively on stable microtubules, with the former being found in dendrites and the latter largely in axons (Bernhardt and Matus, 1984; Dehmelt and Halpain, 2005). The well-studied AD-associated Tau protein in healthy neurons binds to and promotes microtubule polymerization and stability (Weingarten et al., 1975; Cleveland et al., 1977; Panda et al., 2003). A recent study reported a novel function of Tau as a recruiter of EB proteins to microtubule plus-ends, further suggesting, that Tau might contribute to microtubule stability at the +end via EB proteins (Sayas et al., 2015). Mutational studies have shown that MAPs are important for maintaining neuronal morphology. For example, loss of MAP1A shows decreased density of microtubules, abnormal focal swellings in dendrites and degeneration of Purkinje neurons in mice (Liu et al., 2015). MAP1B deficient mice neurons show that this protein sequesters microtubule end-binding proteins EB1 or EB3. This sequestration prevents hyper-stabilization of microtubules and halts axon growth (Tortosa et al., 2013). Thus, interaction of MAPs with microtubules modulates its dynamics and thereby, neuronal function.

## Unstable microtubules in neurodegenerative diseases

Cytoskeletal dysfunctions have been proposed as an underlying mechanism in many neurodegenerative diseases. AD, a widely studied neurodegenerative disease, has been associated with altered microtubule dynamics (Matsuyama and Jarvik, 1989). AD and other tauopathies are characterized by the presence of neurofibrillary tangles (NFTs), composed mainly of hyper-phosphorylated or modified Tau protein (Avila et al., 2004; Iqbal et al., 2010; Serrano-Pozo et al., 2011). Tau also contributes to neuronal morphology and axon outgrowth (Weingarten et al., 1975; Cleveland et al., 1977; Harada et al., 1994; Dawson et al., 2001; Panda et al., 2003; Yu et al., 2008; Sayas et al., 2015). Tau-mediated neurodegeneration could occur via defective axonal transport, faulty signaling or altered gene regulation and may not occur exclusively through microtubule effects (Trinczek et al., 1999; Shulman and Feany, 2003; Blard et al., 2007; Guthrie et al., 2009; Ambegaokar and Jackson, 2011; Morris et al., 2011; McCormick et al., 2013). Nonetheless, some pathological features in Tau-associated diseases could be a consequence of decreased microtubule stability.

A characteristic feature associated with AD is the accumulation of aluminum (Matsuyama and Jarvik, 1989; Walton, 2013) which, when incorporated in microtubules, make them more sensitive to depolymerization (Walton, 2006, 2009). The late onset of AD symptoms can also be attributed to the gradual accumulation of aluminum to levels toxic enough to tip the balance toward microtubule depolymerization and thus possibly neuron degeneration. Similarly, Parkin, an E3 ubiquitin ligase linked to PD, strongly binds to α/β Tubulin heterodimers and stabilizes microtubules (Ren et al., 2003; Yang et al., 2005). Reduction in neurite length, number of neurite branches and synaptic terminals, seen in PD patients, has been attributed to increased microtubule depolymerization in the absence of functional Parkin (Ren et al., 2015). Early loss of neurites, with delayed damage to the soma or “dying back axonopathy,” is a common feature of several neurodegenerative diseases (Stokin et al., 2005; Goto et al., 2009; Dadon-Nachum et al., 2010). Studies in vertebrates and invertebrates show that disruption of pre-synaptic microtubules precedes synapse retraction and degeneration (Zhai et al., 2003; Luo and O'Leary, 2005; Pielage et al., 2005; Stephan et al., 2015). Neurotoxins that lead to Parkinsonism, such as MPP+ (Cappelletti et al., 2005) and 6-hydroxydopamine (Patel and Chu, 2014), also alter microtubule dynamics, causing a decrease in length and number of microtubules and shortening of neurite. Reduction in microtubule stability also underlies the behavioral and axonal transport defects seen in PD-associated mutations in Leucine Rich Repeat Kinase 2 (LRRK2) (Godena et al., 2014). These examples suggest, that decreased stability of microtubules is a common feature in many neurodegenerative diseases and could initiate “dying-back” of the axon (Figure 2B).

## Hyper-stabilized microtubules in neurodegeneration

Although a major fraction of microtubules in differentiated neurons is stable, pools of dynamic microtubules undergoing polymerization and depolymerization are also present (Hu et al., 2008). Stable microtubule pools may be important for maintaining neuronal morphology and synaptic integrity (Lewcock et al., 2007; Jaworski et al., 2009); however, dynamic pools within a neuron are important for turnover of microtubules as well as regeneration of neurons after injury (Stone et al., 2010; Chen and Chisholm, 2011; Ghosh-Roy et al., 2012). Microtubules stabilized using taxol, in healthy neurons, are known to increase the axonal diameter, enlarge growth cones and reduce neurite extension (Letourneau et al., 1986; Chuckowree and Vickers, 2003; Ferrari-Toninelli et al., 2008) (Figure 2C). Taxol stabilizes microtubules and can arrest mitosis and induce apoptosis (Jordan and Wilson, 1998). Therefore, it has been used as an effective treatment against cancer cells (Klauber et al., 1997; Jordan and Wilson, 1998; Dumontet and Sikic, 1999). However, taxol and other microtubule-stabilizing agents used as anti-cancer drugs show adverse effects on the peripheral nervous system in clinical studies. Such drugs have been shown to induce degeneration and fragmentation of sensory axons, reduce axonal length and also reduce axonal transport in animal models (Cavaletti et al., 1995; Lee and Swain, 2006; Scripture et al., 2006; Scuteri et al., 2006; Gornstein and Schwarz, 2014).

Genetic mutations in the microtubule-severing enzyme Spastin, is most commonly associated with hereditary spastic paraplegia (HSP) (Hazan et al., 1999). Spastin loss of function results in local accumulation of detyrosinated microtubules (Tarrade et al., 2006) and reduced number of dynamic plus-ends marked by EB3 along the axon shaft (Fassier et al., 2013). These mutations also results in axonal swellings which can be rescued by treatment with microtubule-destabilizing drugs such as Nocodazole (Fassier et al., 2013). This hyper-stability of microtubules likely triggers the progressive degeneration of corticospinal tracts in the Spastin-dependent HSP cases (Hazan et al., 1999; Evans et al., 2005). Collectively these studies demonstrate that reduced dynamics of microtubules is also detrimental to neuronal health.

## Microtubule dynamics is necessary for neuronal function

Many neurodegenerative diseases are also associated with memory loss (Sagar et al., 1988; Levy et al., 2002; Walsh and Selkoe, 2004; Yamasaki et al., 2007; Shankar et al., 2008). Behavioral paradigms that lead to memory formation also show increase in dynamic microtubules that allows for the necessary synaptic plasticity (Gu et al., 2008; Jaworski et al., 2009; Fanara et al., 2010). Likewise, stabilizing microtubules using taxol prevents memory formation in mice (Atarod et al., 2015). One early symptom in AD is impairment in memory formation while loss of long-term memory appears in more advanced stages of the disease (Selkoe, 2002; Celone et al., 2006). These studies suggest that disruption of microtubule dynamics could be an important parameter, leading to an inability to form new memories as seen in the early stages of some neurodegenerative diseases.

## Perspective: Microtubule dynamics and cellular lingchi?

Given the critical role of microtubules in a variety of cellular processes in the neuron, several of which have been described above, it is not very surprising that this polymer shows changes in multiple neurodegenerative diseases such as AD, PD, Charcot Marie Tooth (CMT) etc. (Evans et al., 2005; Bunker et al., 2006; Gillardon, 2009; Tanabe and Takei, 2009; Cartelli et al., 2010; Tischfield et al., 2010). Amongst the many changes in microtubules that can occur, we think that change in dynamics is a significant “intermediate” phenotype that should be investigated. Microtubule dynamics, and conversely their stability, probably result from the sum of multiple microtubule binding proteins and modifications of α and β-Tubulins. The number of stable and dynamic microtubules has the potential to change a variety of steps (hence Lingchi) including the number of signaling hubs, morphology of the neuron as well as transport rates (Figure 2). Such changes could cumulatively lead to irreversible effects over the time scales of years, often the typical progression time scales in several neurodegenerative diseases.

Recognizing the importance of microtubule dynamics as an intermediate step in disease progression, some studies have focused on increasing stability of microtubules as a therapeutic target in treating neurodegenerative diseases. Axonal transport has been improved by stabilizing or destabilizing microtubules in some models of neurodegenerative disease (Fanara et al., 2007; Cartelli et al., 2010; Zhang et al., 2012; Fassier et al., 2013) and has been suggested as a therapeutic intervention (Ballatore et al., 2012; Brunden et al., 2014). Additionally, targeting Notch signaling as a microtubule stabilizer has also been proposed as therapeutic treatment for neurodegenerative conditions (Bonini et al., 2013). These studies suggest that stabilizing microtubules will improve disrupted axonal transport that could be critical in neurodegenerative disease progression.

However, what appears essential for neuronal function, is not merely stable microtubules but the right balance between stable and dynamic microtubules. This likely impacts numerous cellular processes, not restricted merely to axonal transport (Figure 2). For example, during neuronal polarization, a single neurite, which would be the future axonal process, shows increased microtubule stability (Witte et al., 2008). Moreover, shifting the dynamics toward greater stability by application of taxol results in multiple axons (Witte et al., 2008). The ratio of stable to dynamic microtubules also appears to be critical in allowing neurons to maintain their synaptic plasticity and form new memories (Jaworski et al., 2009; Fanara et al., 2010). In mouse models of neurodegenerative diseases like CMT, it was shown that the disease pathology has two distinct temporal phases, with an early pre-symptomatic phase showing hyper-stable microtubules followed by a later symptomatic phase, where microtubules are unstable (Almeida-Souza et al., 2011a,b; d'Ydewalle et al., 2011). This suggests that one way to slow down disease progression could be to provide temporally defined, correct dosage of agents that control the balance of stable and dynamic microtubules in a disease-specific manner. Additionally, systematic temporal studies examining microtubule dynamics in both cell and animal models, combined with behavioral assays in animal models, may allow investigators to gain a deeper understanding of how the complex cellular roles of microtubules contribute to neurodegenerative disease progression.

### Conflict of interest statement

The authors declare that the research was conducted in the absence of any commercial or financial relationships that could be construed as a potential conflict of interest.
