# Corrigendum: Neurodegeneration and microtubule dynamics: death by a thousand cuts

**DOI:** 10.3389/fncel.2016.00026

**Published:** 2016-02-09

**Authors:** Jyoti Dubey, Neena Ratnakaran, Sandhya P. Koushika

**Affiliations:** ^1^Department of Biological Sciences, Tata Institute of Fundamental ResearchMumbai, India; ^2^Institute for Stem Cell Biology and Regenerative MedicineBangalore, India; ^3^Manipal UniversityManipal, India

**Keywords:** Microtuble stability, Alzheimer's disease, Parkinson Disease, dying back, hyperstable microtubules, microtubule signaling hubs

In the original manuscript, an affiliation for Jyoti Dubey as Manipal University was missing. In addition we would prefer to write expanded name of InStem as Institute for Stem Cell Biology and Regenerative Medicine. This correction does not affect scientific integrity of the review.

## Author contributions

All authors listed, have made substantial, direct and intellectual contribution to the work, and approved it for publication.

### Conflict of interest statement

The authors declare that the research was conducted in the absence of any commercial or financial relationships that could be construed as a potential conflict of interest.

